# Single live cell TGF-β signalling imaging: breast cancer cell motility and migration is driven by sub-populations of cells with dynamic TGF-β-Smad3 activity

**DOI:** 10.1186/s12943-015-0309-1

**Published:** 2015-02-22

**Authors:** Rodney B Luwor, Dulani Hakmana, Josephine Iaria, Thao V Nheu, Richard J Simpson, Hong-Jian Zhu

**Affiliations:** Department of Surgery (RMH), The University of Melbourne, The Royal Melbourne Hospital, Parkville, VIC 3050 Australia; Department of Biochemistry, La Trobe Institute for Molecular Science, La Trobe University, Melbourne, VIC 3086 Australia

**Keywords:** TGF-β, Smad3, Metastasis

## Abstract

**Background:**

Metastasis is a process where only a small subset of cells is capable of successfully migrating to and propagating at secondary sites. TGF-β signalling is widely known for its role in cancer metastasis and is associated with cell migration in whole cell populations.

**Findings:**

We extend these findings by investigating the role of TGF-β signalling in promoting migration and motility by imaging the signalling activity in live, individual MDA-MB-231 cancer cells utilizing a novel Smad3 Td-Tomato reporter adenovirus. Here we find that not all MDA-MB-231 cancer cells have similar TGF-β mediated Smad3 transcription activity and display at least two distinct migratory populations. Importantly, Smad3 activity was significantly higher within migratory cells compared to non-migrated cells in wound healing and transwell assays. Furthermore, time-lapse experiments showed that MDA-MB-231 cells displaying Smad3 activity moved faster and a greater distance compared to cells not displaying Smad3 reporter activity. Interestingly, despite being more motile than cells with undetectable levels of Smad3 activity, high Smad3 activity was detrimental to cell motility compared to low and medium level of Smad3 activity.

**Conclusions:**

We have developed a method enabling real-time visualization of TGF-β signalling in single live cells. Breast cancer cell motility and migration is driven by sub-populations of cells with dynamic TGF-β-Smad3 activity. Those sub-populations may be responsible for tumor invasion and metastasis.

**Electronic supplementary material:**

The online version of this article (doi:10.1186/s12943-015-0309-1) contains supplementary material, which is available to authorized users.

## Background

Tumour metastasis is a multistep process with each phase presenting physical, biological and immune challenges that must be overcome by metastatic capable tumour cells to achieve successful propagation of the secondary site [1,2]. Subsequently, only a very small percentage of tumour cells successfully metastasize [3]. However, the critical molecular differences between this small subset of cells compared to their non-metastatic neighbors within the same tumour population have not been fully established. Furthermore, clinical evaluation of metastasis generates overall genomic and proteomic differences between primary versus secondary tumours following metastatic completion and thus does not assess the dynamic changes during each stage of metastasis [4-6]. Successful movement through each stage of metastasis is dependent upon tumour cells possessing a favorable pro-metastatic signalling signature and is paramount upon appropriate timing of activity of these signalling networks, where activity may be critical for one phase but inhibitory for another.

Transforming Growth Factor-β (TGF-β) regulates a plethora of cellular processes including promotion of cancer invasion and metastasis [7-10]. Many studies have shown that TGF-β increases pro-invasive factors, enhances migration and invasion in culture [11-14] and drives metastasis in animal models [15-17]. However, these observations have been made by assessing the effect of TGF-β across the global, overall tumour population. We expand on these findings in this current study to determine the effect of real-time TGF-β signalling while tracking live single cell movement.

## Results and discussion

### Cells with increased TGF-β signalling activity exhibit enhanced wound healing

**Figure 1:**
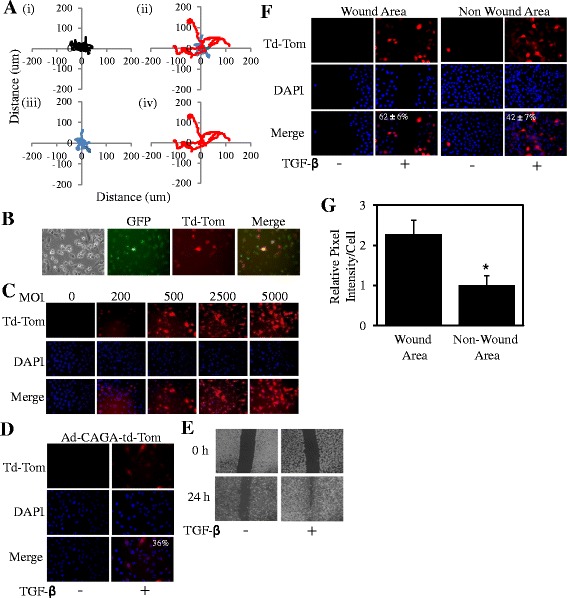
**Live single cell TGF-β signalling promotes wound healing. A**. MDA-MB-231 cells were treated without (i) or with (ii) TGF-β (5 ng/ml) then tracked for 10 h with images taken every 4 min. Slow moving cells (iii) and fast moving cells (iv) within the same cell population treated with TGF-β. **B**. MDA-MB-231 cells were infected with Ad.Cre-GFP and Ad.CAGA-Td-Tom virus, stimulated with TGF-β and imaged for both GFP and Td-Tom. **C**. MDA-MB-231 cells were infected with Ad.CMV-Td-Tom at varying MOI or **D**. Ad.CAGA-Td-Tom virus at a MOI of 2500. Following stimulation with ± TGF-β (5 ng/ml) for 24 h, cells were fixed, permeabilised and stained with DAPI. Percentage positivity was calculated by visualising Tomato expression (Red) compared to nuclear staining (blue). **E**. Wound Area at 0 and 24 h post wound after cells had been infected with Ad.CAGA-Td-Tom virus and stimulated with ± TGF-β (5 ng/ml). **F**. 24 h post wound, cells were fixed, permeabilised and nuclear stained as above and images were taken visualizing Smad3 active cells (red) and nuclear staining (blue). 42% (360 out of 867) cells were positive in the non-wound area versus 62% (175/279) cells in the wounded area. **G**. The relative pixel intensity of Smad3 activity was quantified (Average of 5 randomly chosen fields ± SD). These data are representative of at least 3 separate experiments (*P < 0.05).

Time-lapse microscopy studies showed that MDA-MB-231 cells (human breast carcinoma cell line) moved an overall greater distance when stimulated by TGF-β (Figure 1A(ii)) compared to without (Figure 1A(i)). Intriguingly, we observed two distinctive motility patterns within the whole population of cells stimulated with TGF-β, less motile cells (Figure 1A(iii)) and more motile cells (Figure 1A(iv)). This result was seen consistently across at least 3 separate experiments. This led us to determine if individual cells displayed varied TGF-β-driven Smad3 activity within the same population. This was achieved by engineering an adenoviral Smad3-Td-Tomato reporter (*Ad.CAGA-Td-Tom*) that yields Td-Tomato fluorescence intensity directly proportional to the level of Smad3 transcriptional activity. Td-tomato expression increased as expected in both a TGF-β dose-dependent (Additional file 1: Figure S1A, B) and time-dependant manner (Additional file 1: Figure S1C) similarly to that expected with Smad3 activity. Importantly, our adenovirus did not change any observable properties of TGF-β stimulation, as cells infected with the Ad-CAGA-Td-Tom virus still observed expected, well established outcomes of TGF-β-driven Smad2/3 activity including enhanced Smad2 nuclear localisation despite Adenoviral infection (Additional file 1: Figure 1D). To determine whether our adenovirus could be infected into every cell, cells were co-infected with *Ad-Cre-GFP* and *Ad.CAGA-Td-Tom* prior to TGF-β stimulation (Figure 1B). Every cell was infected as evident by all cells showing detectable GFP, while Td-Tomato was only detected in TGF-β-responsive cells. To further confirm that our adenovirus was entering every cell (and therefore is a true indication of Smad3 activity within every cell), we used a Multiplicity of Infection (MOI) that produced Td-Tomato expression in 100% of MDA-MB-231 cells when driven by a CMV promoter (Ad.CMV-Td-Tom) (Figure 1C). At this MOI (2500), we observed that approximately 36% of MDA-MB-231 cells displayed detectable Smad3 transcriptional activity after 24 h of TGF-β stimulation, compared to 0% without TGF-β (Figure 1D). We have consistently seen a plateau of approximately 40% of TGF-β/Smad3 driven td-Tomato positive cells across a range of MOI’s (Additional file 2: Figure S2). Likewise, Td-Tomato expression driven by the CMV promoter was observed in 100% of U87MG human glioblastoma cells at an MOI of 2500 (Additional file 3: Figure S3A). At this MOI, approximately 5% of U87MG cells displayed detectable Smad3 reporter activity after infection of the Ad.CAGA-Td-Tom virus (Additional file 3: Figure S3B). These results are consistent with previous reports where Smad3 phosphorylation is often observed in heterogeneous patterns throughout clinical or mouse tumour sections indicating that not all cells within a tumour are uniformally active for TGF-β-Smad signalling at any one time [13,18-21].

We next infected MDA-MB-231 cells with the Ad.CAGA-Td-Tom adenovirus in a wound healing assay. TGF-β could clearly accelerate the overall movement of MDA-MB-231 cells into the wound area (Figure 1E) and significantly enhanced Smad3 activity in cells within and outside the wound area compared to unstimulated cells (Figure 1F). Importantly, we found that a significantly greater percentage of cells (62 ± 6%) in the wound area displayed Smad3 activity compared to the non-wound area (42 ± 7%) after TGF-β stimulation (Figure 1F). Furthermore, the relative pixel intensity of Smad3 activity per cell in the wound area was also significantly 2-fold higher compared to the intensity in cells from the non-wound area (Figure 1G). A greater percentage of Smad3 active cells were seen in the wound area (14.9%) compared to the non-wound area (4%) in the U87MG cell line also (Additional file 3: Figure S3C). These results indicate that TGF-β-induced Smad3 activity is more prevalent in cells that are capable of “closing” the wound and may suggest that Smad3-active cells are more motile compared to their non-Smad3 active counterparts within the same MDA-MB-231 population.

### Cells with increased TGF-β signalling activity exhibit enhanced cell migration

**Figure 2:**
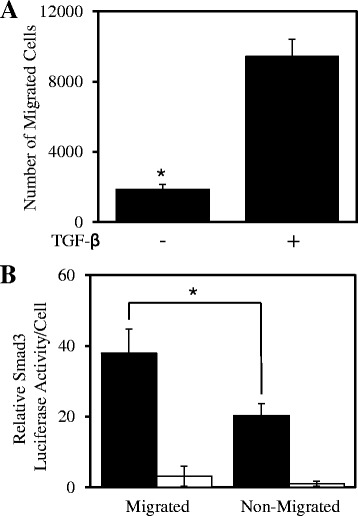
**TGF-β signalling promotes cell migration.** MDA-MB-231 cells were infected with Ad.CAGA-Fluc and Ad.CMV-Gluc virus then stimulated with ± TGF-β (5 ng/ml) before being seeded onto the apical chamber of transwells and a 96-well plate in triplicate for 48 h. Cells on the underneath of the transwell membrane and in the 96-well plate were lysed. **A**. The number of migrated cells through the transwell membrane was calculated as described in the Materials and Methods (Average ± SD). These data are representative of 3 separate experiments (*P < 0.05). **B**. Lysates were then assessed for Smad3 luciferase activity per cell with TGF-β stimulation (■) or without (□) and normalized with CMV-driven Gluc activity (Average ± SD).

Similarly to wound healing, TGF-β stimulation promotes cell migration and invasion [14,22]. To confirm these findings in our system and examine Smad3 activity in migrating cells we co-infected MDA-MB-231 cells with the Smad3-driven and CMV-driven luciferase adenoviruses, Ad.CAGA-Fluc and Ad.CMV-Gluc and performed transwell migration assays. As non-migrated cells are difficult to collect we seeded cells into a 96-well plate as a model for non-migratory cells. Direct assessment of cell number revealed that TGF-β enhanced transwell migration approximately 4-fold compared to cells without TGF-β stimulation (Figure 2A). Furthermore Smad3 activity in migratory cells was significantly 2-fold greater compared to non-migratory cells (Figure 2B). However, these results only measure the average TGF-β-Smad3 signalling activity across the global cell population.

**Figure 3:**
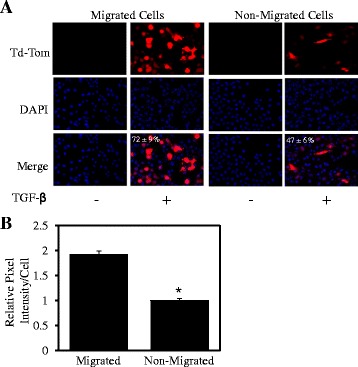
**Live cell TGF-β signalling promotes cell migration.** MDA-MB-231 cells were infected with Ad.CAGA-Td-Tom virus and stimulated with ± TGF-β (5 ng/ml) before being seeded in the apical chamber of transwells and a 24-well plate in triplicate for 48 h. Cells on the underneath of the transwell membrane and in the 24-well plate were fixed, permeabilised and stained with DAPI. **A**. Images were taken of the cells underneath the membrane (migrated cells) and the 24-well plate (non-migrated cells) using a fluorescent microscope visualizing Smad3 active cells (red) and nuclear staining (blue). These data are representative of 3 separate experiments. **B**. The relative pixel intensity of Smad3 activity was quantified using imageJ software and presented as the average of 3 randomly chosen fields ± SD; (n = 3; *P < 0.05).

Therefore to determine whether this overall TGF-β-mediated pro-migratory response was due to specific migratory cells displaying greater TGF-β signalling activity, we infected MDA-MB-231 cells with our Ad.CAGA-Td-Tom adenovirus and performed transwell assays. As above, cells were seeded into a tissue culture plate as a model for non-migratory cells. Indeed, we found that a significantly greater percentage of cells (72 ± 9%) that had migrated through the transwell membrane displayed Smad3 activity compared to the non-migrating cells (47 ± 6%) after TGF-β stimulation (Figure 3A). Furthermore, the relative pixel intensity of Smad3 activity per cell in the migratory cells was significantly 2-fold higher compared to the intensity seen in cells from the non-migratory sub-group (Figure 3B). These results suggest that TGF-β signalling may be switched on during cell migration. Our data is consistent with a previous report by Giampieri and colleagues who showed that transient activation of TGF-β-Smad3 signalling was present in single metastatic cells moving from the primary site. Interestingly, they showed that lung colonisation required down-regulation of TGF-β signalling therefore suggesting that TGF-β signalling activity within metastatic capable cells must fluctuate from high to low for successful metastasis [15].

### Cells with increased TGF-β signalling activity exhibit enhanced cell movement

**Figure 4:**
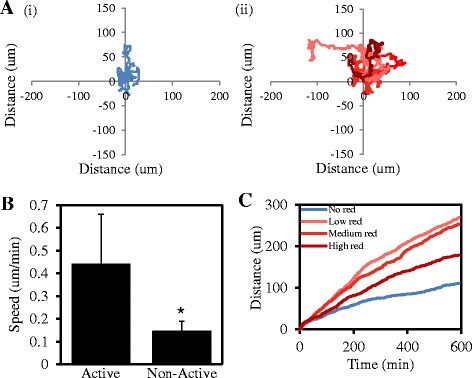
**TGF-β Signalling within live individual tumour cells promotes cell motility. A**. MDA-MB-231 cells were infected with Ad.CAGA-Td-Tom virus and stimulated with ± TGF-β (5 ng/ml). 24 h later, cells were tracked for 10 h and imaged every 4 min. Images were quantified with the use of Metamorph software, to track the distance moved by individual cells with (i) undetectable Smad3 activity and (ii) Smad3 activity following infection with Ad.CAGA-Td-Tom virus and TGF-β stimulation (n = 10). **B**. Average Speed (um/min) of each group of 10 cells (*P < 0.05). **C**. Cells displaying Smad3 activity were divided into 3 categories based on the intensity (low, medium and high) of Smad3 activity. Data represent the average distance travelled over time from each group (n = 7).

We next determined whether active TGF-β-Smad3 signalling correlated with greater cell movement in real time. Live MDA-MB-231 cells were tracked by time-lapse microscopy after infection with the Ad.CAGA-Td-Tom adenovirus and TGF-β stimulation. As observed in Figure 1, TGF-β-induced Smad3-driven Td-Tomato expression in some, but not all cells. Importantly, cells with detectable Smad3 activity moved a greater average distance (Figure 4A(ii)) compared to those that did not (Figure 4A(i)). In addition, Smad3 active cells moved significantly faster (um/min) compared to non-active cells (Figure 4B). Interestingly, the average rate of movement was highest in the cells expressing low and medium intensity Smad3 reporter activity followed by cells expressing high intensity (Figure 4C). A recent study found that cells that were heterozygous for Smad3 were more invasive then wild-type cells and a Smad3^+/−^ mouse contained significantly greater incidence of metastasis than wild-type mice [13]. Although Smad3 activity was not correlated to Smad3 allele number, these and our results indicate that an intermediate level of TGF-β signalling may be more favourable for tumour cell motility and metastasis compared to higher Smad3 activity. Our current data identifies increased TGF-β-driven Smad3 activity specifically in the subset of cells which migrate and are more motile within the overall population. It is predicted that these cells are the most likely to metastasis in a biological system and thus our findings are significant as understanding the key signalling events in the metastatic capable cells allows for a greater understanding in how to inhibit these cells. Attempts to target specific signalling molecules within live cells with greater metastatic potential may provide cancer patients with enhanced therapeutic benefit compared to the clinical outcomes currently achieved.

## Conclusions

TGF-β is only one critical pro-migratory signalling network with combinational signals from other pathways and most likely the dynamic timing of these signals orchestrating successful tumour metastasis. Our adenoviral reporter system provides proof-of-principle evidence that the duration and intensity of signalling molecules/pathways can be examined in individual cells corresponding to cell behavior and may be achieved examining two or more signalling pathways within the one cell population. Importantly, our system may allow in vivo examination of a signalling pathway of interest such as the TGF-β network through each dynamic step of metastasis specifically focusing on the critical small subset of metastatic capable cells.

## Methods

### Cell lines and cell culture

The human malignant breast carcinoma cell line, MDA-MB-231 and the human glioblastoma cell line U87MG were purchased from ATCC (American Type Culture Collection) and were maintained in DME medium containing 10% heat inactivated foetal bovine serum, 2 mM glutamine, 100 μg/ml penicillin and 100 μg/ml streptomycin. Cells were incubated in 10% CO_2_ at 37°C in a humidified incubator. All experiments were performed in DME media containing 10% foetal bovine serum.

### Immunofluorescence staining and confocal microscopy

MDA-MB-231 cells were seeded onto coverslips in 6-well plates and allowed to adhere overnight. Cells were stimulated with or without TGF-β for 15 min. Cells were then washed twice in PBS, fixed in formaldehyde and permeabilized with PBS containing 0.2% Triton-X-100. Following blocking in PBS-Tween20 containing 5% BSA, cells were stained with anti-Smad2 antibody. Visualisation was achieved with Alexa^488^-conjugated secondary antibody using confocal microscopy as described [21,23].

### Generation of the Ad-CAGA-Td-Tom Smad3 reporter and CMV-driven Td-Tom adenoviruses

The *pCAGA*_*12*_*-luc* DNA which specifically measures Smad3 activity [24] was first cloned into the *pENTR 1A* entry clone vector (Invitrogen) to generate pENTR-CAGA-luc as previously described [25]. The td-Tomato gene from *pCMV-Td-Tom* (clonetech) was PCR amplified using forward primers containing EcoRI and HindIII restriction sites and reverse primers containing EcoRI and XbaI restriction sites and cloned into pENTR 1A (Invitrogen) to yield pENTR-Td-Tom. Subsequent cloning of the td-Tomato gene into pENTR-CAGA-luc using restriction enzymes HindIII and XbaI was then performed such that the luciferase gene was removed, yielding pENTR-CAGA-Td-Tom. LR recombination was then performed with the *pAd/PL-DEST* Destination vector (Invitrogen) to generate the p*Ad-CAGA*_*12*_*-Td-Tom* Adenoviral plasmid. The plasmid was digested with *Pac I* and then transfected into the adenovirus producing 293A cell line using Lipofectamine LTX transfection reagent (Invitrogen). Cells were harvested approximately 2 weeks after transfection when lysis was observed in the majority of cells. The adenovirus was amplified, titred and used to detect TGF-β responses and Smad3 activity. Similarly, Td-Tomato was cloned from pENTR-Td-Tom into the *pAd/CMV/V5-DEST* Destination vector (Invitrogen) using EcoRI restriction enzymes to generate the p*Ad.CMV-Td-Tom* Adenoviral plasmid. The p*Ad.CMV-Td-Tom* adenovirus was then produced and amplified as outlined above.

### Multiplicity of infection (MOI) experiment

MDA-MB-231 and U87MG cells were seeded on to a 12 well tissue culture plate and infected with either *Ad.pCMV-td-tomato virus* or *Ad.pCAGA-td-tomato virus* at varying MOI (0, 200, 500, 2500 and 5000) and stimulated with or without TGFβ1 (5 ng/ml). After 48 h the cells were washed with PBS, fixed by adding 3.7% formaldehyde (Sigma-Aldrich, NSW, Australia) for 10 min and permeabilised by adding 0.2% Triton X-100 in PBS. A nuclear stain was performed by adding DAPI (1.5 μg/ml) for 10 min and washed with PBS followed by a DDW wash. The cells were then imaged using a fluorescent microscope. A minimum of 100 cells were counted to calculate the percentage of tomato positive cells (driven by CMV and CAGA) and the intensity of both CMV and CAGA driven tomato expression was determined using image J software.

### Wound healing assay

Cells were infected with *Ad.pCAGA-td-tomato virus* at an MOI of 2500 and seeded on to a 6 well tissue culture plate. 24 h later cells were stimulated with or without TGFβ1 (5 ng/ml). 48 h after seeding the cells, an in vitro wound was produced by scratching the cell monolayer using a sterile 200 μl pipette tip, the media was aspirated and new media with or without TGF-β (5 ng/ml) was added. The plates were left in a 37°C incubator for 5 min and imaged using a phase contrast microscope. 24 h after the scratch, the same fields were imaged. The cells were washed with PBS and fixed by adding 3.7% formaldehyde for 10 min and permeabilised by adding 0.2% Triton X-100 in PBS. A nuclear stain was performed by adding DAPI (1.5 μg/ml) for 10 min and washed with PBS followed by a DDW wash. The cells were then imaged using a fluorescent microscope. The images were quantified using image J software where the total amount of cells and the amount of cells expressing CAGA driven tomato expression (CAGA tomato reporter activity) in the wound area and non-wound area were counted. The intensity of the CAGA tomato reporter activity was also calculated using Image J software.

### Transwell tomato assay

The basal chamber of the transwell was pre-treated with or without TGFβ1 (5 ng/ml) for 1 h. Cells were infected with *Ad.pCAGA-td-tomato virus* at a MOI of 2,500 and treated with or without TGF-β (5 ng/ml). 25,000 cells/well were seeded in to the apical chamber of the transwell and in to a 24 well plate in triplicate. Following 48 h incubation, cells on the top surface of the membrane were wiped off with cotton buds dipped in PBS and discarded. The cells in the 24 well plate and the bottom surface of the membrane were washed with PBS and fixed by adding 3.7% formaldehyde for 10 min and permeabilised by adding 0.2% Triton X-100 in PBS. A nuclear stain was performed by adding DAPI (1.5 μg/ml) for 10 min and washed with PBS followed by a DDW wash. Cells were then imaged using a fluorescent microscope and the percentage of CAGA tomato reporter active cells and the intensity of the CAGA tomato reporter activity was measured using Image J software.

### Transwell luciferase assay

The basal chamber of the transwell was pretreated with or without TGFβ1 (5 ng/ml) for 1 h. Cells were infected with *Ad.pCAGA-td-luciferase virus* and *Ad.Gaussia-td-luciferase virus* at a MOI of 2500 and treated with or without TGF-β (5 ng/ml). 25,000 cells/well were seeded in to the apical chamber of the transwell in triplicate and 2,500 cells were seeded in to a 96 well plate in triplicate. Following 48 h incubation, cells on the top surface of the membrane were wiped off with cotton buds dipped in PBS and discarded. The membranes were peeled off the insert and placed in empty wells. Cells were then lysed and assessed for luciferase activity using the Luciferase Reporter Assay Kit (Roche, NSW, Australia) following the manufacturer’s instructions. Firefly luciferase activity was normalized by the Gaussia luciferase readings and the CAGA luciferase reporter activity per cell was calculated. The number of cells which migrated was calculated by using the cell number in the 96 well plate and the Gaussia luciferase readings using the following calculation: Number of cells transferred through transwell = gaussia reading of bottom chamber × cell number/gaussia reading of 96 well plate.

### Time lapse experiment

Cells were infected with or without *Ad.pCAGA-td-tomato virus* at a MOI of 2500 and seeded on to a 6 well tissue culture plate at 20% confluency and stimulated with or without TGF-β (5 ng/ml). 24 h later, cells were imaged every 4 min for 10 h under 10x objective at 37°C and 5% CO_2_ (Nikon eclipse Ti-E). The images were analyzed using Metamorph software version 7.7.9.0 (Molecular divisions, USA) to calculate the distance that the cells moved. The cells were tracked for up to 10 h (600 min) to calculate the distance (μm) moved. The cells with Smad3 driven tomato expression were divided into low red, medium red and high red groups according to the intensity of their CAGA tomato reporter activity.

### Statistical analysis

All statistical analysis performed using a two-tail students’ *T*-Test (p < 0.05 indicating a statistical significance).

## Additional files

Additional file 1: Figure S1. Smad3-Td-Tomato fluorescence is proportional to the Smad2/3 Activity. MDA-MB-231 cells were infected with Ad.CAGA-Td-Tom virus and **A**, **B**. stimulated with ± TGF-β (0-10 ng/ml) or **C**. TGF-β (5 ng/ml) for various time point. Cells were fixed, permeabilised and stained with DAPI, nuclear staining (blue). The relative pixel intensity of Smad3 activity (Tomato fluorescence, red) was quantified (Average of 5 randomly chosen fields ± SD). These data are representative of at least 3 separate experiments (*P < 0.05). **D**. MDA-MB-231 cells were infected with Ad.CAGA-Td-Tom virus and stimulated with ± TGF-β (5 ng/ml). Cells were then fixed, permeabilised and stained for Smad2 expression and visualized with Alexa488-conjugated secondary antibody (green) and td-Tomato expression using confocal microscopy. Additional file 2: Figure S2. MDA-MB-231 cells were infected with *Ad.pCAGA-td-tomato adenovirus* at the multiplicity of infections (MOI) indicated and stimulated with or without TGFβ1 (5 ng/ml). Cells were then fixed, permeabilised and stained with DAPI. Percentage positivity was calculated by visualizing Tomato expression (red) compared to total cells (blue). Additional file 3: Figure S3. Single Cell TGF-β Smad3 Activity in U87MG cells Promotes Wound Healing. **A**. U87MG cells were infected with Ad.CMV-Td-Tom at varying MOI or **B**. Ad.CAGA-Td-Tom virus at a MOI of 2500. Following stimulation with ± TGF-β (5 ng/ml) for 24 h, cells were fixed, permeabilised and stained with DAPI. Percentage positivity was calculated by visualising Tomato expression (Red) compared to nuclear staining (blue). **C**. 24 h post wound, U87MG cells were fixed, permeabilised and nuclear stained as above and images were taken visualizing Smad3 active cells (red) and nuclear staining (blue).

## Abbreviations

TGF-β: Transforming growth factor-beta
ATCC: American type tissue collection
MOI: Multiplicity of infection
Ad: Adenovirus
Fluc: Firefly luciferase
Gluc: Gaussia luciferase
DMEM: Dulbecco’s modified Eagle's medium
FBS: Foetal bovine serum
