# Supplementary figures and images for: Single live cell TGF-β signalling imaging: breast cancer cell motility and migration is driven by sub-populations of cells with dynamic TGF-β-Smad3 activity
Source: Mol Cancer. 2015 Feb 22;14:50. doi: 10.1186/s12943-015-0309-1 (PMC4343191; doi:10.1186/s12943-015-0309-1)

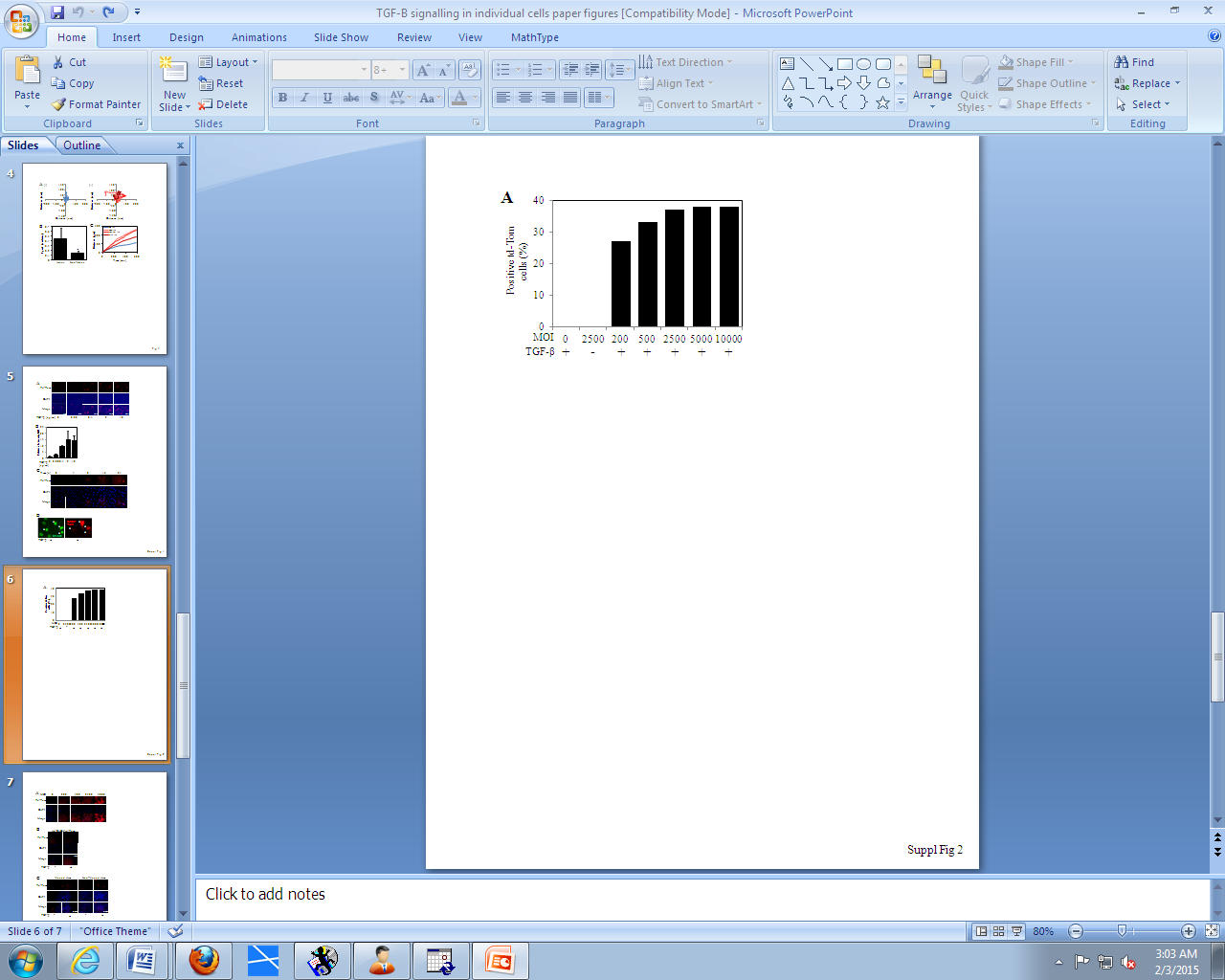

Supplement: Additional file 2: Figure S2. — MDA-MB-231 cells were infected with Ad.pCAGA-td-tomato adenovirus at the multiplicity of infections (MOI) indicated and stimulated with or without TGFβ1 (5 ng/ml). Cells were then fixed, permeabilised and stained with DAPI. Percentage positivity was calculated by visualizing Tomato expression (red) compared to total cells (blue). [file 12943_2015_309_MOESM2_ESM.doc]

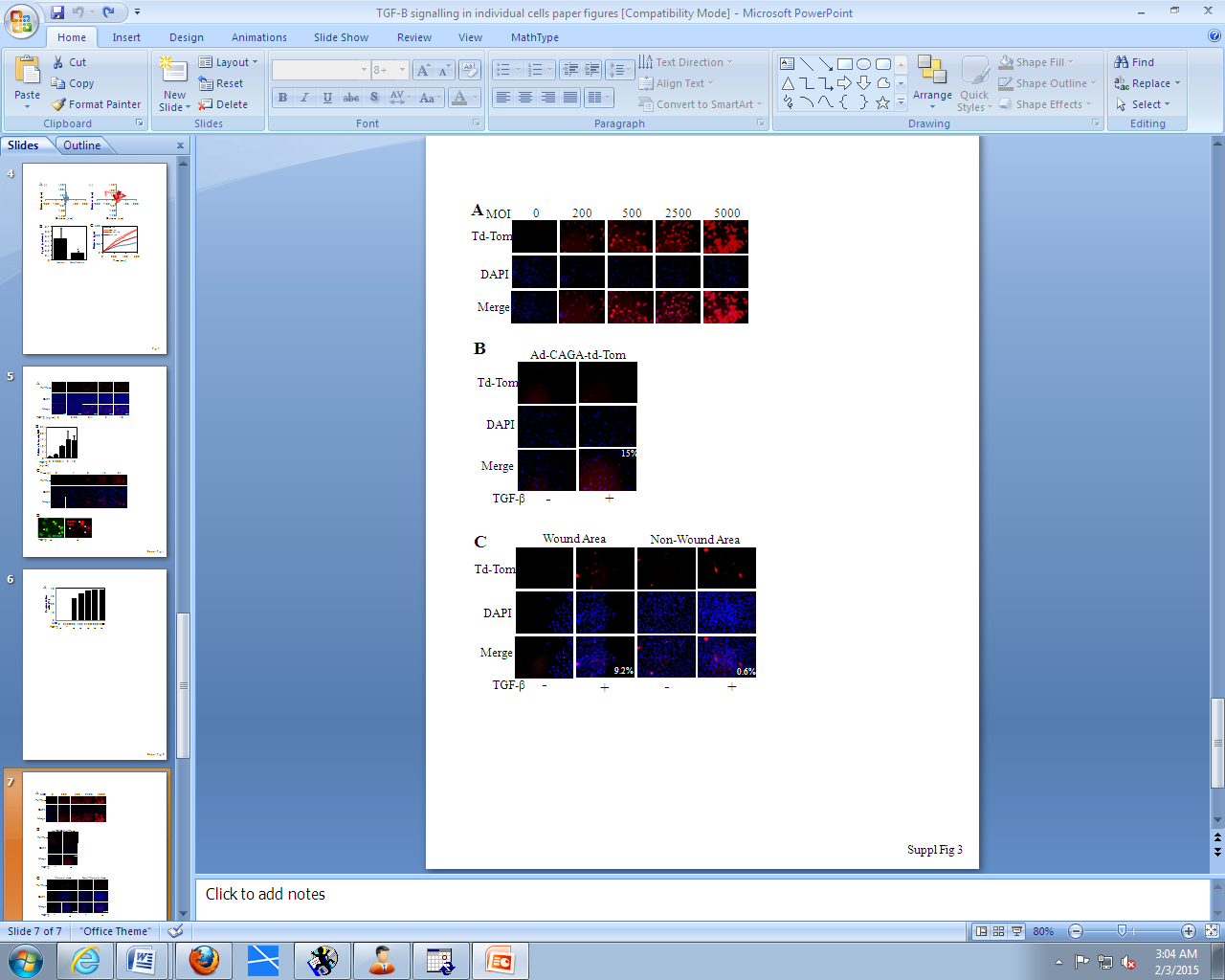

Supplement: Additional file 3: Figure S3. — Single Cell TGF-β Smad3 Activity in U87MG cells Promotes Wound Healing. A. U87MG cells were infected with Ad.CMV-Td-Tom at varying MOI or B. Ad.CAGA-Td-Tom virus at a MOI of 2500. Following stimulation with ± TGF-β (5 ng/ml) for 24 h, cells were fixed, permeabilised and stained with DAPI. Percentage positivity was calculated by visualising Tomato expression (Red) compared to nuclear staining (blue). C. 24 h post wound, U87MG cells were fixed, permeabilised and nuclear stained as above and images were taken visualizing Smad3 active cells (red) and nuclear staining (blue). [file 12943_2015_309_MOESM3_ESM.doc]
