# Supplementary material for: Single live cell TGF-β signalling imaging: breast cancer cell motility and migration is driven by sub-populations of cells with dynamic TGF-β-Smad3 activity
Source: Mol Cancer. 2015 Feb 22;14:50. doi: 10.1186/s12943-015-0309-1 (PMC4343191; doi:10.1186/s12943-015-0309-1)
Supplement: Supplementary file 2 [file 12943_2015_309_MOESM1_ESM.doc]

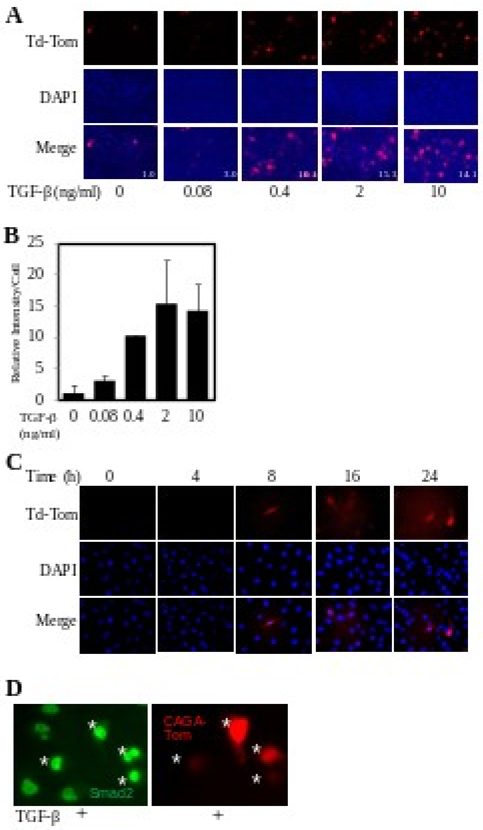
A
Td-Tom
DAPI
Merge
TGF-β(ng/ml) 0 0.08 0.4 2 10
B
25
20
15
10
5
0
Relative Intensity/Cell
TGF-β (ng/ml) 0 0.08 0.4 2 10
C
Time (h) 0 4 8 16 24
Td-Tom
DAPI
Merge
D
Smad2
CAGA-Tom
TGF-β + +

Suppl Fig 1
